# Adult Neurogenesis 50 Years Later: Limits and Opportunities in Mammals

**DOI:** 10.3389/fnins.2016.00044

**Published:** 2016-02-19

**Authors:** Luca Bonfanti

**Affiliations:** ^1^Neuroscience Institute Cavalieri OttolenghiOrbassano, Italy; ^2^Department of Veterinary Sciences, University of TurinTorino, Italy

**Keywords:** neural stem cells, brain repair, parenchymal progenitors, astrocytes, comparative neurogenesis, mammalian brain

After five decades of research in adult neurogenesis (AN) it is far from easy to make a balance. If this field was a movie genre, brain repair goals would be a dreary mystery (with cell replacement therapies approaching fantasy), opportunities would be high quality science fiction, and limits could well belong to a hopeless thriller. Though apparently depicting a pessimistic screenplay, these aspects actually represent very exciting plots in which the only pitfall had been the attitude of those main characters (the scientists) who, starting with the re-discovery of AN (Paton and Nottebohm, 1984; Lois and Alvarez-Buylla, 1994), looked for neuronal cell replacement. The chimera of regenerative outcomes led to an exponential burst of studies: more than 7500 articles on PubMed with the keyword “adult neurogenesis.” Why such an interest many years after the first demonstration of AN (Altman and Das, 1965)? Maybe because the first isolation of neural stem cells (NSCs) took place in the same period (Reynolds and Weiss, 1992), thus making it possible to figure out continuous replenishment of new neurons throughout a brain's life (Gage, 2000; Alvarez-Buylla et al., 2001). At the same time, the possibility to play *in vitro* with the NSC plasticity (Galli et al., 2003) might explain why the AN articles in PubMed become 23,000 when the keyword “neural stem cell” is employed.

## Revisiting the history of AN

**Figure 1:**
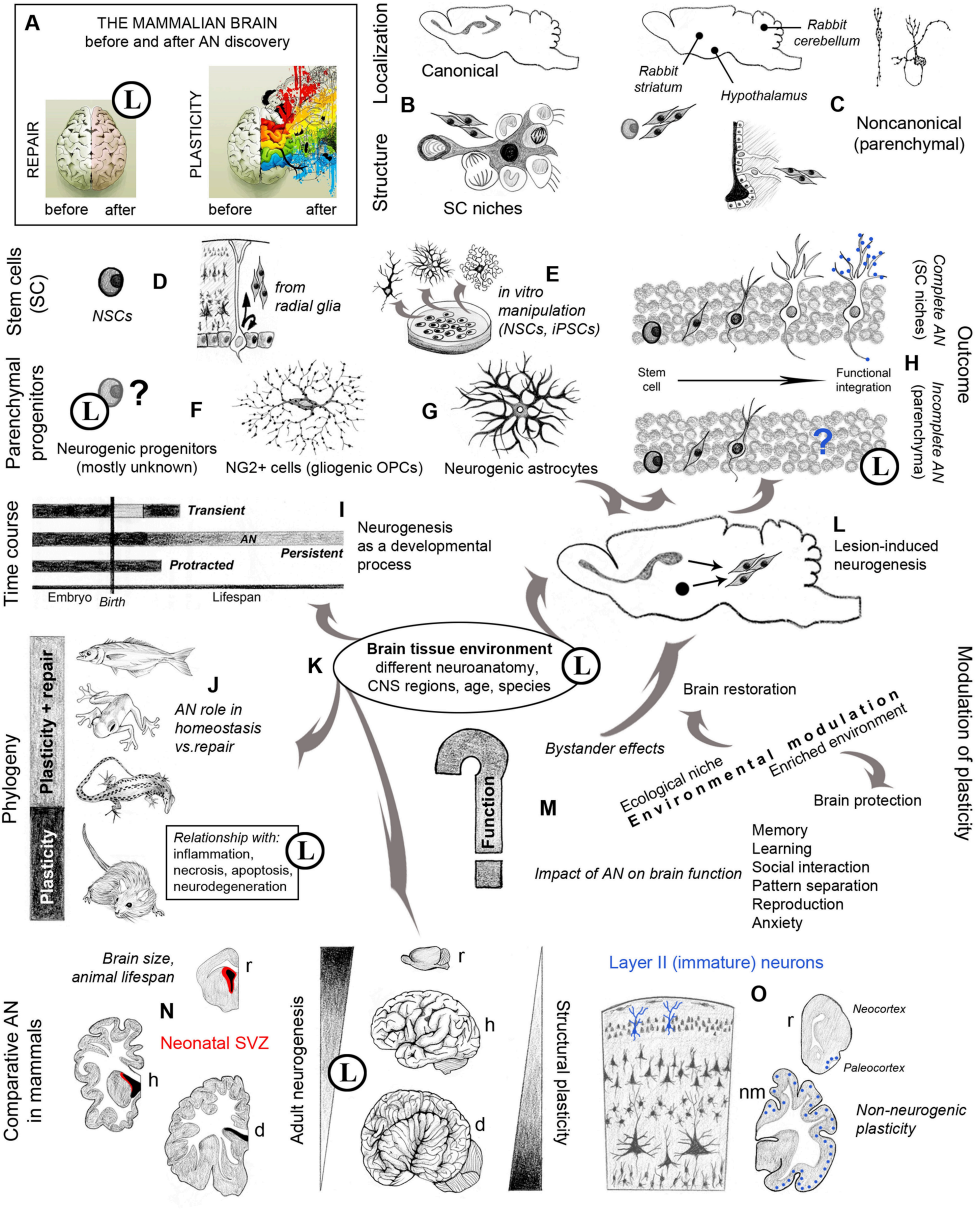
**Graphic representation of multifaceted aspects and new opportunities arisen in neurobiology by the study of adult neurogenesis (AN)**. Circled L indicate when substantial limits are also present. **(A)** An image originally referring to brain hemisphere asymmetry (http://thebiointernet.org/training-of-right-brain-hemisphere-and-intuitive-information-sight-in-bratislava/) is used here to represent the new vision of brain plasticity after AN discoveries; beside remarkable limits still existing in brain repair (pale pink), most opportunities involve new forms of structural plasticity with respect to the old dogma of a static brain (rainbow colors). **(B)** Canonical sites of AN, harboring well characterized stem cell niches (Tong and Alvarez-Buylla, 2014; Vadodaria and Gage, 2014). **(C)** Different types and locations of non-canonical neurogenesis do occur in various brain regions, depending on the species (Luzzati et al., 2006; Ponti et al., 2008; Feliciano et al., 2015). **(D)** NSCs are astrocytes originating from bipotent radial glia cells (Kriegstein and Alvarez-Buylla, 2009); **(E)** the occurrence of stem cells in the brain gives rise to (theoretically endless) in vitro manipulations. **(F)** Parenchymal progenitors are less known; most of them are gliogenic, yet some are responsible for species-specific/region-specific, non-canonical neurogenesis, and some others can be activated after lesion **(G)** (Nishiyama et al., 2009; Feliciano et al., 2015; Nato et al., 2015). **(H)** The outcome of canonical and non-canonical neurogenesis is different, only the former leading to functional integration of the newborn neurons (Bonfanti and Peretto, 2011); blue dots: synaptic contacts between the new neurons and the pre-existing neural circuits. **(I)** Strictly speaking, AN should be restricted to the continuous, “persistent” genesis of new neurons, which is different from “protracted” neurogenesis (delayed developmental processes, e.g., postnatal genesis of cerebellar granule cells, postnatal streams of neuroblasts directed to the cortex; Luzzati et al., 2003; Ponti et al., 2006, 2008), and “transient” genesis of neuronal populations within restricted temporal windows (e.g., striatal neurogenesis in guinea pig; Luzzati et al., 2014). **(L)** Reactive neurogenesis can be observed in different injury/disease states both as a cell mobilization from neurogenic sites and as a local activation of parenchymal progenitors (Arvidsson et al., 2002; Magnusson et al., 2014; Nato et al., 2015). **(J)** Evolutionary constraints have dramatically reduced the reparative role of AN, involving tissue reactions far more deleterious than in non-mammalian vertebrates (Weil et al., 2008; Bonfanti, 2011). **(K)** Failure in mammalian CNS repair/regeneration is likely linked to mature tissue environment, clearly refractory to new neuron integration outside the two canonical NSC niches and relative neural systems; this fact confines AN to physiological/homeostatic roles, which remain undefined in terms of “function.” **(M)** The role of AN strictly depends on the animal species, evolutionary history and ecological niche; its rate and outcome is affected by different internal and external cues; although not being strictly a function, AN can impact several brain functions (Voss et al., 2013; Aimone et al., 2014; Amrein, 2015). **(N)** Different anatomy, physiology, and lifespan in mammals do affect AN rate and outcome; periventricular AN is highly reduced in large-brained mammals (Sanai et al., 2011; Paredes et al., 2015; Parolisi et al., 2015). **(O)** Studies on AN carried out by using markers of immaturity (e.g., DCX and PSA-NCAM) have revealed other forms of plasticity (non-neurogenic), being well represented in large-brained mammals (Gomez-Climent et al., 2008; Bonfanti and Nacher, 2012). r, rodents; h, humans; d, dolphins; nm, non-rodent mammals. Drawings by the Author.

Most AN review articles start with Altman's pioneering studies, disregarded at the time by most neurobiologists and then upgraded to the death of a dogma (Gross, 2000). What is more difficult to find is a critical evaluation of what happened after the nineties. Briefly, an intense phase of AN characterization contributed to persuade the scientific community that stem cells actually persist in the adult mammalian brain (Palmer et al., 1997; Doetsch et al., 1999), making the integration of new neurons a real phenomenon producing anatomical and functional changes (Gage, 2000; Alvarez-Buylla et al., 2001; Lledo et al., 2006). The stem cell niches of two main neurogenic sites (subventricular zone and hippocampal dentate gyrus) were identified and progressively defined in their structure and regulation (Figure 1). On these solid bases, a sort of gold rush-like fever aiming at demonstrating new sites of AN grew exponentially (Gould et al., 1999, 2001; Zhao et al., 2003; Dayer et al., 2005; Shapiro et al., 2007). Yet, some of the “alternative” neurogenic regions were subsequently denied by independent studies (references in Bonfanti and Peretto, 2011; Nacher and Bonfanti, 2015). In parallel, it was shown that neurogenesis can be induced by different types of injury or disease (lesion-induced, reactive neurogenesis), either by mobilization of cells from the neurogenic sites (Arvidsson et al., 2002) or by local activation of parenchymal progenitors (Magnusson et al., 2014; Nato et al., 2015; Figure 1). Nevertheless, though large numbers of neuroblasts can be produced in response to stroke or inflammation (Arvidsson et al., 2002; Ohira et al., 2010; Magnusson et al., 2014; Nato et al., 2015), the mechanisms of such responses as well as the ultimate fate of the newborn cells remain largely unknown, as acknowledged by leading experts in the field (Lindvall and Kokaia, 2015). In addition, only limited spontaneous recovery occurs (Sohur et al., 2006; Bonfanti, 2011) and some promising results published on megahit journals have not been reproduced (Magavi et al., 2000; Nakatomi et al., 2002). Finally, the huge effort for obtaining regenerative outcomes by using exogenous sources of stem/progenitor cells has also led, until now, to scarce results in terms of reliability and effectiveness (Li et al., 2010), although some therapeutic perspectives might come from the use of stem cell-derived dopaminergic cells in Parkinson disease (Barker et al., 2015).

How can we find an explanation for recurrent failures in obtaining cell replacement from AN? Maybe the answer resides in a psychological attitude: the initial burst of optimism affecting scientists with the biased vision that “new neurons equals brain repair” persisted too long under translational pressures, in forgetfulness of a basic fact: the mammalian central nervous system (CNS) evolved to be substantially nonrenewable, relatively hardwired, non-self repairing (Weil et al., 2008). Further proof come from examples of spontaneous “parenchymal” (non-canonical) neurogenesis detectable in other mammals: the outcome of these newly-produced neurons is quite different from that performed in canonical NSC niches (Feliciano et al., 2015) since “transient” neural cells are mostly produced (Gould et al., 2001; Luzzati et al., 2014). More recently, some neurogenic activity has been shown in the hypothalamus, starting from tanycytes harbored within a germinal layer-derived zone, linked with feeding regulation and energy balance, and responding to external stimuli (Migaud et al., 2010). Yet, low levels of neurons are generated in basal conditions, and their final outcome is far from clear.

Hence, if regarding AN as a “full biological process” (from NSC activation to neuronal integration), all neurogenic phenomena occurring out of the hippocampus and olfactory bulb should be classified as “incomplete” (Bonfanti and Peretto, 2011), both spontaneously-occurring and reactive neurogenic events appearing as “unwanted hosts” in the mature brain tissue (Figure 1K).

## The big questions in AN

By putting together data learned over 50 years of AN research with CNS evolutionary history, it appears clear that: (i) AN has lost most of its capacity for brain repair in mammals with respect to other vertebrates (Grandel and Brand, 2013), its role being largely restricted to physiological plasticity of specific systems (Peretto and Bonfanti, 2014); (ii) this feature might not primarily depend on the availability of stem cells (AN does exist in mammals!) rather on CNS structural, cellular, molecular organization, as a result of its postnatal development and immunological responses (Bonfanti, 2011). Hence, one big question concerns the intermix of biological events leading to such a loss of regenerative capacity.

Many scientists working in the field focus on the question: how NSCs divide and regulate their quiescent/active state *in vivo*? (in the perspective of modulating—usually intended as “increasing”—their mitotic activity and neuronal fate). These actually are crucial points in NSC basic biology. Yet, beside the common viewpoint considering the neurogenic potential of NSCs to be beneficial, the fact is emerging that having more new neurons or synapses is not always better (Tang et al., 2014; e.g., hippocampal AN can be implicated in memory erasure, Akers et al., 2014; Kitamura and Inokuchi, 2014). By contrast, I consider as essential questions: whether, how, when different types of progenitor cells can produce a progeny which can actually survive and functionally integrate in the brain regions in which they are needed, out of the two canonical niches. Even within the niches, specific subsets of progenitors occupying precise topographical subregions produce only selected neuronal types for selected tissue domains (Obernier et al., 2014), thus confirming that mature brain neurogenic plasticity occurs only within restricted bounds. Also in gliogenesis, the amount of oligodendrocyte precursor cells (OPCs) generated daily in the adult CNS (Young et al., 2013; Boda and Buffo, 2014) clashes with the slow rate of myelin turnover, suggesting that only a small fraction of them actually integrate. Moreover, they appear able to sustain remyelination after acute lesion or disease but not in chronic phases (Franklin, 2002).

A fundamental issue regards the molecular and cellular features which make the mature mammalian brain environment refractory to substantial reshaping or repair, both in physiological and pathological states, with respect to the permissive conditions existing in non-mammalian vertebrates (Kyritsis et al., 2014; Figures 1J,K). Unfortunately, the tools at present available to address such aspect are scarce. One possible way could reside in neurodevelopmental studies aimed at unraveling how the embryonic, permissive tissue environment shifts to mature, more restrictive conditions (Peretto et al., 2005), taking into account that a regulated balance of stability and plasticity is required for optimal functioning of neuronal circuits (Abraham and Robins, 2005; Akers et al., 2014). This approach could open new landscapes from the re-expression of developmental programs (Sohur et al., 2012) to the cutting edge frontier of homeosis (Arlotta and Hobert, 2015).

Another fundamental question remains substantially unanswered (and often skipped by scientists hurrying in search for reparative roles of AN): concerns the function of AN (Figure 1M). It seems clear that AN can play a physiological role in memory and learning, yet rapid adaptation of hippocampal neurogenesis to experimental challenges appears to be a characteristic of laboratory rodents, whereas low or missing AN in bats and dolphins argues against a critical role in spatial learning (Amrein and Lipp, 2009). Wild mammals show species-specific, rather stable hippocampal neurogenesis, which appears related to demands that characterize the niche exploited by a species rather than to acute events in the life of its members (Amrein, 2015). It is worthwhile to remember that AN itself should not be considered as a “function,” rather a tool the brain can use to perform different functions (see also Hersman et al., 2016). As stated by Anderson and Finlay (2014), “Mounting evidence from allometric, developmental, comparative, systems-physiological, neuroimaging, and neurological studies suggests that brain elements are used and reused in multiple functional systems.” They suggest that “this variable allocation can be seen in neuroplasticity over the life span,” and that “the same processes are evident in brain evolution (interaction between evolutionary and developmental mechanisms to produce distributed and overlapping functional architectures in the brain).” That is to say: brain evolution is an ultimate expression of neuroplasticity, and more systematic information about evolutionary perspectives is needed to set out the question of the normal functionality of new neurons.

## Astrocytes and other, widely ramified, opportunities

The most counterintuitive discovery in half a century of AN research concerned the central role of astrocytes as primary progenitors for neuron production (Alvarez-Buylla et al., 2001). Across the years, new roles for these glial cells progressively emerged in different steps of the AN process, from maintenance of the NSC niche, through substrate for migration and functional integration of the newlyborn neurons (Sultan et al., 2015), to that of parenchymal progenitors activated by lesion (Magnusson et al., 2014; Nato et al., 2015). The regional and temporal heterogeneity of astrocytes should be among the big issues for future investigation of brain plasticity (Bayraktar et al., 2015), but this is only one example indicating how deeply different is our vision of brain structure and function before and after AN discovery. More recent breakthroughs concern the modulatory effects of lifestyle on AN (e.g., how exercise protects and restores the brain; Voss et al., 2013), and many emerging roles of the new neurons in impacting brain functions such as social interaction, reproduction, memory, learning, pattern separation, overgeneralization of sensory stimuli, and anxiety disorders (Leuner and Gould, 2010; Sahay et al., 2011; Feierstein, 2012; Kheirbek et al., 2012; Figure 1M). Furthermore, a vast range of “bystander effects” acting through paracrine or immunemodulatory mechanisms can exert beneficial effects by modifying the microenvironment at the injury site through the release of chemokines/cytokines (Martino et al., 2011; Kokaia et al., 2012; Pluchino and Cossetti, 2013). Other ramifications involve the big chapter of widespread gliogenesis (Nishiyama et al., 2009), whose effects are not limited to glial cell renewal, since bystander functions are also emerging for OPCs (Boda and Buffo, 2014; Birey et al., 2015). Yet, in the complex intermix of interactions involved in AN, most processes remain ill-defined as “ghost outcomes” of the stem cell activity (including the transient existence of the progeny), thus being worthwhile of further investigation.

Finally, unexpected trends are emerging from comparative studies showing how the spatial and temporal extent of AN dramatically decreases in large-brained, long-living species (e.g., humans and dolphins; Sanai et al., 2011; Parolisi et al., 2015; Patzke et al., 2015) with respect to small-brained, short-living rodents (Paredes et al., 2015; Figure 1N). The use of markers usually expressed in newly born neurons (e.g., doublecortin) led to reveal the existence of immature, non-newly generated cells (Gomez-Climent et al., 2008) which are more abundant in large-brained species (Luzzati et al., 2009; Bonfanti and Nacher, 2012; Figure 1O). This fact opens new hypothesis about the evolutionary choices in terms of structural plasticity among mammals, again underlining the importance of comparative studies (Lindsey and Tropepe, 2006; Bonfanti et al., 2011).

## Conclusion

Even if we are still far from healing most brain lesions and neurodegenerative diseases, we have gained a fully new vision of brain plasticity (Figure 1A). In AN history, it seems that scientists have made serious sins in their approach. Yet, there are many reasons for forgiveness linked to the extremely innovative character of their work aimed at unraveling the dynamic nature of a brain tissue constrained within limits of invariability imposed by evolution. Five decades after the first demonstration of AN we still need to place it in the domain of basic research aimed at unraveling cellular, molecular, and evolutionary aspects of an extremely complex biological process. Maintaining a substantial independence from translational pressures (what implies hard work of teaching the values of fundamental research to grantmakers) could lead to higher achievements: the understanding of brain function and plasticity.

Looking back to its origin and forward to its future, the AN research field is maybe one of the best movies ever shot in the neurosciences, with passion and love for the unknown prevailing at the beginning of the story, then gradually shifting to magical realism toward the end.

## Author contributions

The author confirms being the sole contributor of this work and approved it for publication.

### Conflict of interest statement

The author declares that the research was conducted in the absence of any commercial or financial relationships that could be construed as a potential conflict of interest.
